# Inhibitory effects of bisbenzylisoquinolines on synthesis of the inflammatory cytokines interleukin-1 and tumour necrosis factor-alpha

**DOI:** 10.1155/S0962935193000262

**Published:** 1993

**Authors:** W. Kim Seow, Kazuhiro Nakamura, Yukio Sugimura, Yukihiro Sugimoto, Yasuyuki Yamada, David P. Fairlie, Y.H. Thong

**Affiliations:** 1 lmmunobiology Laboratory The University of Queensland Dental School Brisbane 4000 Australia; 2 Tochigi Research Laboratories Kao Corporation 2606 Akabane Ichikai-machi, Tochigi 321-34 Japan; 3 Department of Agricultural Chemistry Faculty of Agriculture Kyoto University Kyoto 606 Japan; 4 Centre for Drug Design and Development The University of Queensland Brisbane 4072 Australia; 5 Immunobiology Laboratory The University of Queensland Mater Children's Hospital South Brisbane 4001 Australia; 6 Arid Land Research Center Tottori University 1390 Hamasaka Tottori 680 Japan

## Abstract

Synthesis of IL-1β and TNFα by human monocytesmacrophages was significantly inhibited by eleven bisbenzylisoquinolines and one half-molecule (benzylisoquinoline), with IC_50_ values in the μM range. The results indicate that these compounds may have value in the therapy of human diseases where these inflammatory cytokines have a central role in pathogenesis.


Mediators of Inflammation, 2, 199–203 (1993)

**SYNTHESIS of IL-1β and TNFα by human monocytes-macrophages was significantly inhibited by eleven bisbenzylisoquinolines and one half-molecule (benzylisoquinoline), with $IC_{50}$ values in the μM range. The results indicate that these compounds may have value in the therapy of human diseases where these inflammatory cytokines have a central role in pathogenesis.**

**Key words:** Bis-benzylisoquinolines, Cytokines, IL-1β, TNF-α

### Inhibitory effects of bisbenzylisoquinolines on synthesis of the inflammatory cytokines interleukin-1 and tumour necrosis factor-alpha

**W. Kim Seow,[1] Kazuhiro Nakamura,[2] Yukio Sugimura,[2] Yukihiro Sugimoto,[*,2] Yasuyuki Yamada,[3] David P. Fairlie[4] and Y.H. Thong.[5,CA]**

[1]Immunobiology Laboratory, The University of Queensland Dental School, Brisbane, 4000, Australia;
[2]Tochigi Research Laboratories, Kao Corporation, 2606 Akabane, Ichikai-machi, Tochigi 321-34, Japan;
[3]Department of Agricultural Chemistry, Faculty of Agriculture, Kyoto University, Kyoto 606, Japan;
[4]Centre for Drug Design and Development, The University of Queensland, Brisbane, 4072, Australia;
[5]Immunobiology Laboratory, The University of Queensland, Mater Children's Hospital, South Brisbane, 4001, Australia
**Present address:* Arid Land Research Center, Tottori University, 1390 Hamasaka, Tottori 680, Japan

[CA] Corresponding Author

### Introduction

The pro-inflammatory cytokines interleukin-1 (IL-1) and tumour necrosis factor (TNF) are potent molecular mediators with wide-ranging effects on a large array of cells, tissues and organs.[1,2] They are produced by a wide variety of cell types, but monocytes-macrophages are the major source of these polypeptides which share structural similarities and have overlapping activities.[3,4] They have been implicated in the pathogenesis of chronic inflammatory (e.g. rheumatoid arthritis[5]), autoimmune (e.g. insulin-dependent diabetes mellitus[6]), allergic (e.g. asthma[7]), infectious (e.g. leprosy[8]) and malignant (e.g. ovarian carcinoma[9]) diseases.

There is therefore a great interest in drugs that can control their generation and/or action because of the potential for therapeutic application. It has recently been shown that tetrandrine, a novel bisbenzylisoquinoline alkaloid, has potent inhibitory effects on the production of IL-1β and TNFα by human monocyte-macrophage cultures.[10–12] In addition, it has been shown to have therapeutic efficacy in a number of animal models of human disease, such as relapsing experimental allergic encephalitis in rats (multiple sclerosis),[13] spontaneous diabetes in BB rats (insulin-dependent diabetes mellitus),[14] air-pouch inflammation in rats (rheumatoid arthritis)[15] and airways microvascular leakage in guinea-pigs (asthma).[16] In an attempt to gain some mechanistic insight into the relationship between the bisbenzylisoquinoline structure and the extent of inhibition of the synthesis of TNF-α and IL-1β, the inhibitory potencies of tetrandrine and a series of ten other bisbenzylisoquinolines and one benzylisoquinoline (half-molecule) are now compared. The bisbenzylisoquinolines vary in the type of substituent, location of their ether bridges and the stereochemistry at a chiral centre. The sole benzylisoquinoline tested is a monomeric component of the dimeric benzylisoquinoline, tetrandrine,

© 1993 Rapid Communications of Oxford Ltd and serves to identify whether the dimeric bisbenzylisoquinolines are active as a result of their inherent macrocyclic structure or because of their constituent functional groups which are also present in their smaller monomeric fragments.

### Materials and Methods

*Compounds:* Four types of compounds were used in these experiments (Fig. 1). Three of these were macrocyclic ether compounds of isoquinolines bridged by alpha-benzyloxy substituents. These compounds differ in the position of the linkages between the two monomeric benzylisoquinoline components, and the nature and number of substituents on the aromatic rings. The fourth type of compound was represented by *N*-methylcoclaurine, a monomeric benzylisoquinoline.

The twelve compounds used in this study were obtained from diverse sources.[17] Tetrandrine and fangchinoline, were extracted from the tubers of *Stephania tetrandra*,[18] which were purchased from Maruzen Seiyaku Company, Onomichi, Japan; cepharanoline, cepharanthine, isotetrandrine and cycleanine, extracted from the tuberous root of *Stephania cepharantha*,[19] were purchased from Kaken Shoyaku Company, Tokyo, Japan; berbamine and oxyacanthine were purchased from Carl Roth, Germany; aromoline was isolated from root cultures of *Stephania cepharantha*, as described previously;[20] homoaromoline and obaberine were converted from aromoline and oxyacanthine, respectively, by methylation with diazomethane; *N*-methylcoclaurine was prepared by chemical synthesis.[21]

All compounds (>97% pure) were identified for chemical structure by mass spectroscopy and $^1$H-NMR spectroscopy against reference samples and literature values.[22] The HCl salts of the compounds were dissolved in RPMI 1640 medium at a concentration of 2 mg/ml, and these stock solutions further diluted in RPMI 1640 medium for the experiments.

*Production of IL-1β and TNFα:* The study was performed with monocytes–macrophages isolated from human blood.[10–12] Blood was drawn by venepuncture from three healthy non-smoking adults not on any medication for at least 2 weeks prior to the study. The heparinized blood was layered onto Hypaque–Ficoll medium of density 1.114 and centrifuged at 600 × *g* for 30 min. The

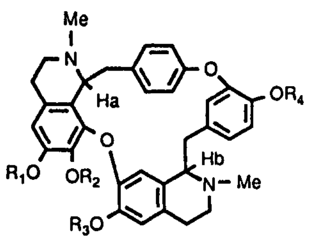

TYPE A

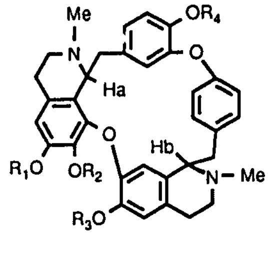

TYPE B

| Derivative | $R_1$ | $R_2$ | $R_3$ | $R_4$ | Ha | Hb |
|---|---|---|---|---|---|---|
| Aromoline | Me | H | Me | H | R | S |
| Homoaromoline | Me | H | Me | Me | R | S |
| Oxyacanthine | Me | Me | Me | H | R | S |
| Obaberine | Me | Me | Me | Me | R | S |
| Cepharanoline | -$CH_2$- | | Me | H | R | S |
| Cepharanthine | -$CH_2$- | | Me | Me | R | S |

| Derivative | $R_1$ | $R_2$ | $R_3$ | $R_4$ | Ha | Hb |
|---|---|---|---|---|---|---|
| Tetrandrine | Me | Me | Me | Me | S | S |
| Fangchinoline | Me | H | Me | Me | S | S |
| Berbamine | Me | Me | Me | H | S | R |
| Isotetrandrine | Me | Me | Me | Me | R | S |

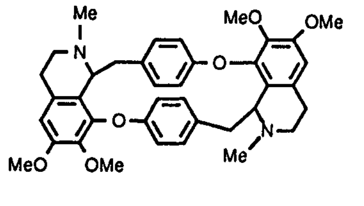

Cycleanine

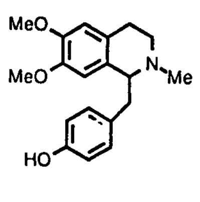

N-Methylcoclaurine

FIG. 1. Chemical structure of the bisbenzylisoquinolines: ten head-to-head conformation (Types A and B), one head-to-tail (cycleanine) and one half-molecule (*N*-methylcoclaurine). Ha and Hb designate absolute configuration (*R* or *S*).

mononuclear leukocytes (MNC) in the top band at the interface were harvested,[23] washed and resuspended in RPMI 1640 medium for the experiments.

Preparation of adherent MNCs, containing predominantly monocytes, were made in sterile plastic Petri dishes. After 3 h at 37°C, the non-adherent cells were washed off, and the adherent cells were later removed with the aid of a sterile rubber policeman, washed and re-suspended in RPMI 1640 medium containing 5% foetal calf serum, and the cell concentration adjusted by counting in a Neubauer haemocytometer. To 1 ml of $2 \times 10^6$ cells/ml was added 0.5 ml of drug solution. After incubation at 37°C for 15 min, 0.5 ml of $4 \times 10^7$ heat-killed, formalin-fixed, *Staphylococcus aureus* as a stimulant, were added into wells of 24-well cluster plates. The supernatants were harvested after 24 h, and kept frozen at −70°C for up to 2 weeks before being assayed for the presence of IL-1$\beta$ and TNF$\alpha$. The cells were checked for viability by Trypan-blue dye exclusion (>94%).

*Quantitation of IL-1β and TNFα:* Both IL-1$\beta$ and TNF$\alpha$ in the culture supernatants were measured by a competitive binding radioimmunoassay (Amersham, UK).[12] The tracer was [$^{125}$I]IL-1$\beta$ or TNF$\alpha$ (human, recombinant), and the antibody to human recombinant IL-1$\beta$ or TNF$\alpha$ was made in rabbits. The antibody-bound IL-1$\beta$ or TNF$\alpha$ was then reacted with the Amerlex-M second antibody reagent (donkey anti-rabbit serum) which contains a second antibody that is bound to magnetizable polymer particles. Separation of the antibody-bound fraction is effected by centrifugation of the Amerlex-M suspension and decantation of the supernatant. Measurement of the radioactivity in the pellet enables the amount of labelled IL-1$\beta$ or TNF$\alpha$ in the bound fraction to be calculated. The concentration of unlabelled IL-1$\beta$ or TNF$\alpha$ in the sample is then determined by interpolation from a standard curve.

*Determination of $IC_{50}$ values:* The concentration of drug ($\mu$M) which causes 50% inhibition of cytokine production ($IC_{50}$) was determined from graphical plots of inhibitor concentration vs. percentage inhibition.

### Results

*Effect on IL-1β and TNFα synthesis:* Human monocytes-macrophages were incubated with 0, 1, 4, 10, 20 and 40 $\mu$g/ml of each of the twelve compounds. The results (Table 1) showed dose dependent inhibition of both IL-1$\beta$ and TNF$\alpha$ production for all twelve compounds. Control values (fM/$10^6$ cells) for quantities of IL-1$\beta$ and TNF$\alpha$ produced by cultures were 162.5 ± 8.2 and 81.2 ± 4.9 (mean ± S.E.M.) respectively, or approximately 2 762 and 1 380 pg/l/$10^6$ cells, respectively.

*$IC_{50}$ values:* The $IC_{50}$ values were measured from graphs of inhibitor concentration vs. % inhibition and are presented in Table 2. The results show that all $IC_{50}$ values for the twelve compounds are within the same order of magnitude and the range 4–26 $\mu$M and 2–18 $\mu$M for IL-1$\beta$ and TNF$\alpha$ respectively.

### Discussion

Currently available drugs for therapy of chronic inflammatory and autoimmune diseases are unsatisfactory because of significant toxic and/or immunosuppressive side effects. There is therefore a great need to develop better and safer drugs to control inflammatory tissue destruction. The discovery of the central role of inflammatory cytokines in the pathogenesis of these diseases[1–9] opens up a new strategic target for drug development. The authors recently showed that the bisbenzylisoquinoline alkaloids, tetrandrine and berbamine, have the capacity to inhibit both the generation and action of IL-1$\beta$ and TNF$\alpha$,[10–12] and control disease progression in a number of animal models.[13–16] Although the mechanism of action of bisbenzylisoquinolines is not well understood, studies with tetrandrine have shown its capacity to block calcium channels,[24] interfere with transmembrane signalling,[25] and induce apoptosis.[26] Moreover, it is largely devoid of immunosuppressive properties,[27,28] and is non-toxic at therapeutic doses (20 mg/kg per day for 84 days) as assessed by appearance, behaviour, weight change, blood chemistry and organ histology of BB rats.[14] This compares well with the oral $LD_{50}$ of 2 230 mg/kg in rats,[29] which is more than 100 times the therapeutic dose. Furthermore, tetrandrine has been used empirically in humans for the treatment of silicosis at a dose of 300 mg/day for 3 years without serious toxic side effects.[30] Thus, bisbenzylisoquinolines may have significant advantages over existing drugs used in the treatment of chronic inflammatory and autoimmune diseases, such as corticosteroids, cytotoxic and anti-rejection drugs, which have substantial toxic and immunosuppressive side effects. As such, they may become the fore-runner of a new class of safe and effective drug suitable for long term clinical usage.

The results of the present study showed that all eleven bisbenzylisoquinoline alkaloids have inhibitory effects on IL-1$\beta$ and TNF$\alpha$ synthesis by human monocytes–macrophages. Calculation of $IC_{50}$ values showed that inhibitory concentrations of these eleven compounds were within the same order of magnitude. This was surprising as the structures depicted in Fig. 1 vary considerably. It was expected

**Table 1**. Effect of bisbenzylisoquinolines on production of IL-1β and TNFα by human monocytes–macrophages

| Drug | Concentration (µg/ml) | Percent of control (±S.E.M.) | | Drug | Concentration (µg/ml) | Percent of control (±S.E.M.) | |
|---|---|---|---|---|---|---|---|
| | | IL-1 | TNF | | | IL-1 | TNF |
| Aromoline | 1 | 89.7 ± 2.9 | 86.0 ± 6.6 | Obaberine | 1 | 95.3 ± 5.7 | 92.6 ± 2.6 |
| | 4 | 71.5 ± 5.2 | 75.3 ± 2.1 | | 4 | 90.4 ± 2.6 | 64.9 ± 6.8 |
| | 10 | 45.7 ± 5.5 | 52.1 ± 12.2 | | 10 | 69.0 ± 3.8 | 30.2 ± 5.1 |
| | 20 | 20.5 ± 7.1 | 39.3 ± 14.2 | | 20 | 45.9 ± 5.6 | 11.3 ± 1.5 |
| | 40 | 8.2 ± 3.4 | 11.7 ± 3.6 | | 40 | 13.1 ± 2.5 | 4.1 ± 0.3 |
| Berbamine | 1 | 87.9 ± 5.1 | 77.3 ± 3.2 | Oxyacanthine | 1 | 101.5 ± 4.8 | 100.3 ± 10.1 |
| | 4 | 83.4 ± 4.8 | 71.5 ± 11.0 | | 4 | 80.9 ± 7.9 | 80.3 ± 10.6 |
| | 10 | 33.7 ± 11.9 | 24.0 ± 12.3 | | 10 | 37.9 ± 12.4 | 32.2 ± 3.9 |
| | 20 | 7.0 ± 0.7 | 3.1 ± 1.3 | | 20 | 16.9 ± 4.2 | 10.5 ± 1.1 |
| | 40 | 4.3 ± 1.0 | 1.5 ± 1.0 | | 40 | 5.6 ± 0.1 | 3.2 ± 1.3 |
| Cepharanoline | 1 | 82.4 ± 7.0 | 63.3 ± 8.8 | Tetrandrine | 1 | 84.3 ± 5.6 | 72.3 ± 8.4 |
| | 4 | 63.9 ± 5.0 | 41.1 ± 13.7 | | 4 | 64.9 ± 4.2 | 39.6 ± 6.3 |
| | 10 | 40.8 ± 10.0 | 23.6 ± 11.0 | | 10 | 16.6 ± 6.0 | 1.2 ± 0.7 |
| | 20 | 23.0 ± 8.8 | 4.5 ± 0.5 | | 20 | 13.0 ± 7.9 | 1.7 ± 1.2 |
| | 40 | 17.1 ± 5.7 | 1.9 ± 0.9 | | 40 | 10.1 ± 6.6 | 0.3 ± 0.0 |
| Fangchinoline | 1 | 94.3 ± 4.9 | 91.7 ± 7.2 | Cepharanthine | 1 | 98.7 ± 11.2 | 53.2 ± 6.3 |
| | 4 | 36.0 ± 6.3 | 44.0 ± 21.2 | | 4 | 84.4 ± 7.7 | 28.7 ± 1.6 |
| | 10 | 3.8 ± 1.0 | 2.4 ± 0.9 | | 10 | 49.8 ± 11.5 | 10.7 ± 0.9 |
| | 20 | 3.0 ± 0.9 | 2.3 ± 0.8 | | 20 | 20.5 ± 5.8 | 3.1 ± 1.1 |
| | 40 | 1.7 ± 0.4 | 1.0 ± 0.6 | | 40 | 5.7 ± 1.1 | 2.1 ± 1.1 |
| Homoaromoline | 1 | 74.3 ± 9.5 | 84.9 ± 6.3 | Cycleanine | 1 | 84.5 ± 3.9 | 74.0 ± 5.0 |
| | 4 | 49.6 ± 8.9 | 66.9 ± 10.7 | | 4 | 78.0 ± 4.2 | 41.2 ± 10.8 |
| | 10 | 36.4 ± 10.8 | 32.4 ± 2.9 | | 10 | 64.0 ± 12.6 | 22.5 ± 10.2 |
| | 20 | 13.6 ± 3.5 | 7.5 ± 1.1 | | 20 | 47.0 ± 14.1 | 12.7 ± 3.5 |
| | 40 | 8.1 ± 3.1 | 2.2 ± 1.56 | | 40 | 36.6 ± 8.5 | 3.3 ± 1.5 |
| Isoterandrine | 1 | 73.9 ± 9.3 | 75.8 ± 6.5 | *N*-methyl-coclaurine | 1 | 92.6 ± 9.5 | 96.6 ± 3.8 |
| | 4 | 70.0 ± 10.3 | 43.2 ± 6.1 | | 4 | 51.8 ± 8.7 | 82.4 ± 12.2 |
| | 10 | 46.5 ± 3.3 | 6.7 ± 1.1 | | 10 | 1.5 ± 3.7 | 15.2 ± 6.3 |
| | 20 | 12.0 ± 4.2 | 1.8 ± 0.7 | | 20 | 1.5 ± 3.0 | 7.7 ± 2.1 |
| | 40 | 5.1 ± 0.8 | 1.0 ± 0.9 | | 40 | 1.6 ± 3.3 | 5.5 ± 1.3 |

The results represent the mean ± S.E.M. of three experiments using cells from three individuals. The quantity (fM/$10^6$ cells) of IL-1β and TNFα produced by control cultures were 162.5 ± 8.2 and 81.2 ± 4.9 (mean ± S.E.M.), respectively.

**Table 2**. Inhibition of IL-1β and TNFα production by bisbenzylisoquinolines: $IC_{50}$ values

| Bisbenzylisoquinolines (HCl salts) | MW | $IC_{50}$ (µM) | |
|---|---|---|---|
| | | IL-1β | TNFα |
| Aromoline | 667 | 13.6 | 18.3 |
| Homoaromoline | 681 | 8.7 | 9.8 |
| Oxyacanthine | 681 | 11.6 | 10.1 |
| Obaberine | 695 | 26.0 | 8.9 |
| Berbamine | 681 | 11.3 | 7.3 |
| Isotetrandrine | 695 | 13.4 | 4.3 |
| Fangchinoline | 681 | 4.6 | 4.6 |
| Tetrandrine | 695 | 7.2 | 4.6 |
| Cepharanoline | 665 | 12.3 | 3.8 |
| Cepharanthine | 679 | 15.0 | 2.2 |
| Cycleanine | 695 | 25.0 | 4.6 |
| *N*-methylcoclaurine* | 335.5 | 11.6 | 16.4 |

* half-molecule that the locations and numbers of methoxy- vs. hydroxy-substituents (types A and B, cycleanine) would have significantly influenced interaction with (unknown) receptors responsible for blocking synthesis of the specific cytokines in question, especially if the dimeric form is maintained intracellularly. Similarly the chirality differences would lead to different molecular orientations of the isoquinolines during interaction with receptor(s). However, the similarity in inhibitor potencies suggested that the dimer structure as such may not be the principal activity determinant.

A very significant finding may be that *N*-methylcoclaurine, a non-cyclic benzylisoquinoline quite different from the cyclic bisbenzylisoquinolines, has equivalent inhibitory effects on IL-1β and TNFα production. There are at least two possible interpretations of these findings in terms of structure–activity relationships. One interpretation would be that the bisbenzylisoquinolines and benzylisoquinolines act on different receptors, even though both are equipotent inhibitors of inflammatory cytokine production. A more likely interpretation is that the monomers, benzylisoquinolines, may be the active forms of the bisbenzylisoquinolines. Bisbenzylisoquinolines like *N*-methylcoclaurine are known to chemically assemble in plants to form bisbenzylisoquinolines like tetrandrine. However, it is not known whether bisbenzylisoquinolines transform biologically into monomeric benzylisoquinolines, although it has been shown chemically that cleavage of tetrandrine can be achieved using strong reducing conditions (metallic sodium in liquid ammonia) into two monomers, one of which is *N*-methylcoclaurine.[31]

One avenue for further investigation would be to determine whether the bisbenzylisoquinolines are metabolized under the conditions of these experiments and, if so, to identify the metabolites after *in vitro* and *in vivo* degradation. Another would be to determine whether bisbenzylisoquinolines and benzylisoquinolines have similar or different molecular modes of actions. Because of the importance of inflammatory cytokines in health and disease, further development of these classes of compounds may provide insights into the biology of inflammatory cytokines as well as to a better and safer drug for therapy of inflammatory diseases.

### References

1. Dinarello CA. IL-1 and its biologically related cytokines. *Adv Immunol* 1989; **4:** 153–205.
2. Balkwill FR, Burke F. The cytokine network. *Immunol Today* 1989; **10:** 299–304.
3. Last-Barney K, Homon CA, Foanes RB, Merluzzi VJ. Synergistic and overlapping activities of tumor necrosis factor-α and IL-1. *J Immunol* 1988; **141:** 527–534.
4. Seow WK, Thong YH, Ferrante A. Macrophage–neutrophil interactions: Contrasting effects of interleukin-1 and tumor necrosis factor-alpha (cachectin) on human neutrophil adherence. *Immunology* 1987; **62:** 357–361.
5. Arend WP, Dayer JM. Cytokines and cytokine inhibitors or antagonists in rheumatoid arthritis. *Arthritis Rheum* 1990; **33:** 305–315.
6. Mandrup-Poulson T. Cytokine-mediated beta-cell destruction: the molecular effector mechanism causing IDDM? *Autoimmunity* 1990; **3** (suppl. 1): 121–122.
7. Brown PH, Crompton GK, Greening AP. Proinflammatory cytokines in acute asthma. *Lancet* 1991; **338:** 590–593.
8. Yamamura M, Uyenmura K. Defining protective responses to pathogens: Cytokine profiles in leprosy lesions. *Science* 1991; **254:** 277–279.
9. Malik S, Balkwill F. Epithelial ovarian cancer: a cytokine propelled disease? *Br J Cancer* 1991; **64:** 617–620.
10. Seow WK, Ferrante A, Li Si-Ying, Thong YH. Suppression of human monocyte interleukin-1 production by the plant alkaloid tetrandrine. *Clin Exp Immunol* 1989; **75:** 47–51.
11. Ferrante A, Seow WK, Rowan-Kelly B, Thong YH. Tetrandrine, a plant alkaloid, inhibits the production of tumour necrosis factor alpha (cachectin) by human monocytes. *Clin Exp Immunol* 1990; **80:** 232–235.
12. Seow WK, Ferrante A, Summors A, Thong YH. Comparative effects of tetrandrine and berbamine on production of the inflammatory cytokines interleukin-1 and tumour necrosis factor. *Life Sci* 1992; **50:** PL53–PL58.
13. Wong CW, Seow WK, Thong YH. Comparative effects of tetrandrine and berbamine on acute and relapsing experimental allergic encephalitis in Lewis rats. *Int Arch Allergy Appl Immunol* 1992; **97:** 31–36.
14. Lieberman I, Lentz PB, Trucco G, Seow WK, Thong YH. Prevention by tetrandrine of the spontaneous development of diabetes mellitus in the BB rat. *Diabetes* 1992; **41:** 616–619.
15. Wong CW, Seow WK, O'Callaghan JW, Thong YH. Comparative effects of tetrandrine and berbamine in the subcutaneous air-pouch model of inflammation. *Agents & Actions* 1992; **36:** 112–118.
16. Wong CW, Thong YH, Seow WK. Comparative effects of tetrandrine and berbamine on guineapig airway microvascular leakage induced by PAF and other allergic mediators. *Int J Immunopharmacol* 1993; **15:** 185–193.
17. Nakamura K, Tsuchiya S, Sugimoto Y, Sugimura Y, Yamada Y. Histamine release inhibition activity of bisbenzylisoquinoline alkaloids. *Planta Medica* 1992; **58:** 505–508.
18. Tomita M, Sheng-Teh Lu. Alkaloids of *Stephania tetrandra* S. Moore. *Yakugaku Zasshi* 1967; **87:** 316–318.
19. Tomita M, Kozuka M. Alkaloids of *Stephania cepharantha* Hayata. *Yakugaku Zasshi* 1967; **87:** 1203–1208.
20. Sugimoto Y, Yamada Y, Sugimura Y. Comparison of bisbenzylisoquinoline alkaloids in root cultures of *Stephania cepharantha* with those of the parent plant. *J Natl Prod* 1989; **52:** 199–202.
21. Kametani T, Yagi H, Kaneda S. A modified total synthesis of stereoisomeric mixture of magnolamine. *Chem Pharm Bull* 1966; **14:** 974–980.
22. Guha KP, Mukherjee B, Mukherjee R. Bisbenzylisoquinoline alkaloids. *J Nat Prod* 1979; **42:** 1–83.
23. Ferrante A, Thong YH. Optimal conditions for simultaneous purification of mononuclear and polymorphonuclear leukocytes from human blood. *J Immunol Methods* 1980; **36:** 109–116.
24. King VF, Garcia ML, Himmel D, *et al.* Interactions of tetrandrine with slowly inactivating calcium channels. Characterization of calcium channel modulation by an alkaloid of Chinese medicinal herb origin. *J Biol Chem* 1988; **263:** 2238.
25. Ioannoni B, Chalmers AH, Seow WK, McCormack JG, Thong YH. Tetrandrine and transmembrane signal transduction: effect on phosphoinositide metabolism, calcium flux and protein kinase C translocation in human lymphocytes. *Int Arch Allergy Appl Immunol* 1989; **89:** 349–354.
26. Teh BS, Chen P, Lavin M, Seow WK, Thong YH. Demonstration of the induction of apoptosis (programmed cell death) by tetrandrine. *Int Arch Allergy Appl Immunol* 1991; **13:** 1117–1126.
27. Li Si-Ying, Teh BS, Seow WK, Ling Li-hua, Thong YH. Effect of tetrandrine on immunological responses and cardiac transplant rejection in mice. *Int Arch Allergy Appl Immunol* 1989; **90:** 169–173.
28. Wong CW, Seow WK, Zeng TS, Halliday WJ, Thong YH. Comparative effects of the bisbenzylisoquinolines tetrandrine and berbamine on immunological responses and experimental brucellosis in mice. *Int J Immunopharmacol* 1991; **13:** 585–589.
29. Chang HM, But PPH. *Pharmacology and Applications of Chinese Materia Medica.* Singapore: World Scientific, 1987.
30. Li Quanlu, Xu Yonghou, Zhou Zeshen. The therapeutic effect of tetrandrine on silicosis. *Chin J Tuberc Respir Dis* 1981; **4:** 321–328.
31. Fujita K, Murai F. Studies on the alkaloids of menispermaceous plants (*Masao Tomiti*). LXXXII. On the structure of biscoclaurine alkaloids (3). Cleavage of tetrandrine by metallic sodium in liquid ammonia. *J Pharmacol Soc Japan* 1951; **71:** 1039–1042.

**Received 11 February 1993; accepted in revised form 5 March 1993**
